# A Meta-Analysis on the Influence of Age-Friendly Environments on Older Adults’ Physical and Mental Well-Being

**DOI:** 10.3390/ijerph192113813

**Published:** 2022-10-24

**Authors:** Jia-Jia Zhou, Rui Kang, Xue Bai

**Affiliations:** 1 Department of Applied Social Sciences, The Hong Kong Polytechnic University, Hong Kong 999077, China; 2 Institute of Active Ageing (Research Centre for Gerontology and Family Studies), The Hong Kong Polytechnic University, Hong Kong 999077, China

**Keywords:** age-friendly environments, meta-analysis, physical well-being, mental well-being, healthy ageing, older adults

## Abstract

The importance of age-friendly environments (AFEs) for older adults has been empirically and theoretically highlighted by the extant literature. However, the strength of the association between environments and older adults’ well-being has not been comprehensively quantified. Given the different attributes of the physical and mental dimensions, this meta-analysis aims to synthesise and quantify the association between AFEs and the physical and mental well-being of older adults. Fourteen eligible studies were included in this analysis: among which eight explored the link between AFEs and physical well-being, and eleven investigated AFEs in association with mental well-being. A random-effects model showed a small but significant correlation between AFEs and the mental well-being of older adults (*r* = 0.160, 95% CI [0.084, 0.224], *p* < 0.001), and the correlation between AFEs and physical well-being was also significant (*r* = 0.072, 95% CI [0.026, 0.118], *p* < 0.01). The number of environmental factors involved in AFEs moderated the association with physical well-being, from which the association was only significant among studies focusing on fewer environmental factors (*n* < 6). Results of this meta-analysis indicated that AFEs may be more effective in promoting the emotions of older adults, compared to ameliorating their physical functioning. The limitations of current empirical studies and directions for future research in the field of environmental gerontology were also discussed.

## 1. Introduction

As a result of the accelerated process of ageing and urbanisation globally, researchers share a growing interest in linking multiple environmental components and a range of well-being outcomes among older adults. As highlighted in the framework for healthy ageing of the World Health Organization (WHO) [1], optimising well-being not only depends on older adults’ intrinsic capacity, but it is also influenced by environmental exposures and their interactions within them. Therefore, creating age-friendly communities and cities is one of the WHO’s priorities in the *Decade of Healthy Ageing: 2020–2030* [2]. Empirical evidence has also captured positive associations between age-friendly environments (AFEs) and older adults’ life satisfaction and quality of life [3,4,5].

However, when focusing on specific aspects of well-being, the influence of environments varies widely across studies. Stephens et al. [6] found that neighbourhood trust and access to facilities are associated with better mental health-related quality of life among older adults in New Zealand, but not with physical health. Different direct effects of liveable environments on both mental and physical health were detected in a study of older adults living in Hong Kong [7]. Studies on super-aged societies, such as Japan, have highlighted the implications on older people’s social and mental well-being from multiple environments (e.g., available information, community services and social participation) [8]. By contrast, the influences of these environmental factors were insignificant or showed a lower correlation among older Chinese adults [9]. Overall, empirical studies present inconsistencies and variations in the association between environments and older adults’ well-being.

A meta-analysis is therefore warranted to provide more conclusive evidence and to address the limitations and mixed findings of earlier studies on the relationship between AFEs and older adults’ physical and mental well-being. The current study’s objectives are to (i) synthesise the research characteristics in extant studies on the relationship between older adults and environment; (ii) identify and quantify whether, and to what extent, combined multiple environmental factors are associated with physical and mental well-being in older adults; and (iii) examine the heterogeneity of the person–environment associations, and to explore potential moderators within this heterogeneity. 

### 1.1. The Concept of Physical and Mental Well-Being 

Promoting well-being is more than a priority for ageing well among older adults [10], it is also a universal social action for global citizens [11]. As a multidimensional construct, the terminology of well-being has been conceptualised, operationalised and measured in various ways [12,13,14,15]. To date, there is no single or universal definition or assessment of well-being. The dissimilar characteristics of the multidimensions of well-being necessitates to analyse the associations between specific aspects of well-being and AFEs separately. In this meta-analysis, we employed the two most frequently inspected dimensions of well-being—physical and mental well-being—to examine their relationship with combined environmental components. Physical and mental well-being were also two key clustered themes in a review of 99 self-reported measures of well-being over two decades [16]. In this way, a more accurate assessment of environmental influences on older adults’ physical or mental well-being could be informed based on the synthesised evidence. 

Specifically, physical well-being refers to the condition and performance of bodily functioning [16]. It includes energy to live independently, the capacity to make use of the external environment, and experiences of feeling physical pain and comfort [16]. Accordingly, measures or indicators of physical well-being among older adults are normally presented as physical performance, functional ability, body pain, frailty, or falls [17,18]. In this meta-analysis, self-rated health was categorised as physical well-being because evidence showed that the measures of physical health (e.g., physical tiredness, physical mobility, chronic disease) contributed more to self-rated health construction [19,20].

Alternatively, guided by the hedonic and eudaimonic perspectives, mental well-being could be generally defined as ‘feeling good’. Concerns related to the dimension of mental well-being reflect the psychological, cognitive, emotional and affective aspects of people’s evaluation of life, and encompass individuals’ thoughts and feelings about the state of their life, and experiences of happiness [16]. In this way, mental well-being is typically assessed by measures such as emotional stability, happiness, life satisfaction, meaning in life, positive emotions, mood, and affection [12,21,22]. Briefly, physical well-being concentrates more on functioning well, while mental well-being emphasises more on individuals’ feelings of contentment; both reflect divergent aspects of well-being in old age. 

### 1.2. Environments in Association with Older Adults’ Well-Being

Theoretically, the association between socio-spatial environments and older adults’ developmental outcomes has been supported by environmental gerontology [23,24]. From the perspective of the press-competence model [25], the ageing process is shaped by the dynamic interaction of environmental press/resources and personal competence. In other words, environmental factors may hinder or constrain older adults’ well-being by blocking or facilitating older adults’ activities or experiences. More recent studies [26,27,28] have tried to explain the person–environment relationship by incorporating more precise processes. They have posited that the process of person–environment belonging and agency effectively link environments to older adults’ community psychology and proactive activities, thus enhancing well-being outcomes (both physical and mental aspects) in later life. 

Previous review studies have examined the influence of environments on various types of well-being from different perspectives. For example, based on the findings of 11 cross-sectional studies, Gong et al. [29] concluded that despite significant heterogeneity, urban environments have a measurable association with psychological distress. Similarly, a narrative review has provided evidence for the protective effects of exposure to natural environments on mental health, cognitive function and physical activity [30]. Another review synthesise neighbourhood safety factors in association with health-related outcomes and showed that general neighbourhood safety is related to older adults’ mental health and physical functioning [31]. However, after fully inspecting 14 studies of randomised controlled trials, Moore et al. [32] discovered a very weak or even no effect of built environment interventions on adults’ mental health and quality of life. Overall, although some review studies have summarised positive associations between environments and people’s physical and mental well-being outcomes, the descriptive conclusions from the narrative approaches are not quantitatively robust. Moreover, not all the review studies focused on the person–environment relationship in older groups, who may be more dependent on their external surroundings in the ageing process. 

This meta-analysis intends to provide a more robust conclusion regarding the association between AFEs and older adults’ physical and mental well-being. Primarily, it aims to determine whether and to what extent multiple environmental factors are correlated with physical and mental well-being. It is worth noting that this meta-analysis did not concentrate on a single environmental factor because AFEs are a comprehensive system in which multiple environmental components are interrelated with each other. In addition, when living in a socio-spatial environment, older adults are inevitably exposed to diverse environmental factors rather than a unidimensional aspect. By uncovering the above-mentioned core question, this meta-analysis will advance our understanding of the relationship between combined environments and the physical and mental well-being of older adults. Research characteristics among selected studies and the heterogeneity of the person–environment associations are also presented. In this way, it may also stimulate research directions or implications for future studies in the field of environmental gerontology. 

## 2. Method

**Figure 1:**
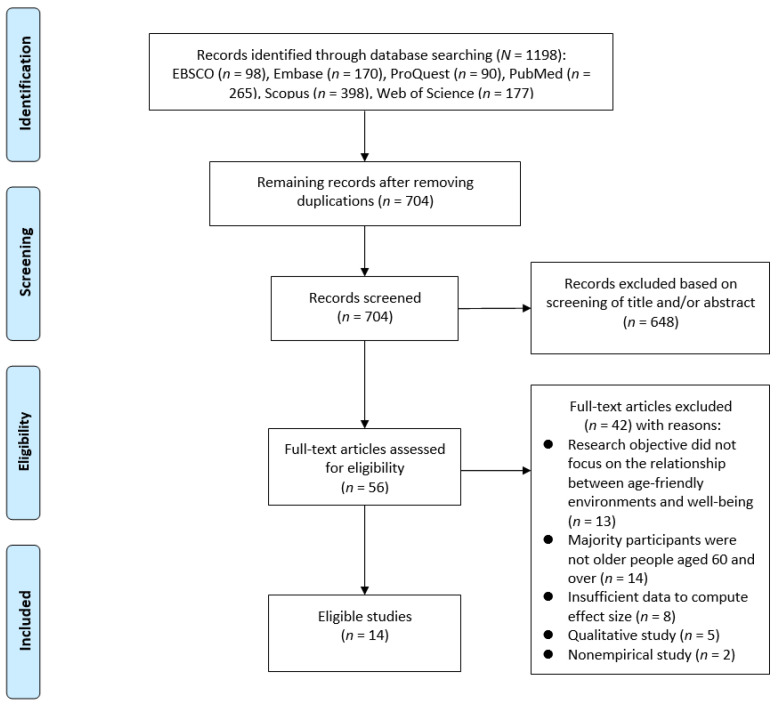
Flow chart of the selection process of meta-analysis.

As shown in Figure 1, this study was conducted according to the guidelines of the preferred reporting items for systematic reviews and meta-analyses (PRISMA) [33]. The selection process was conducted independently by the first and second authors (J.-J.Z. and R.K.). Discrepancies in the selected results for each step were resolved through discussions among the three authors. The dispute resolution in the discussion process was based on the inclusion criteria of studies. The three authors carefully read the studies again and discussed the controversial aspects. Only when all authors agreed that the study satisfied all inclusion criteria could it then be identified as eligible. 

### 2.1. Search Strategy

A systematic search was conducted in five digital academic databases: Scopus, Web of Science, EBSCO, PubMed, and EMBASE. We searched for publications between January 2007 and December 2021 because the concept of an age-friendly community was initially proposed by the WHO in 2007. Key terms concerning outcomes, age-friendly environments, and participants were selected from titles, keywords, and abstracts in the searching process. Three groups of keywords were used: (i) age-friendly environments, age-friendliness, age-friendly communities, age-friendly cities, and age-friendly neighbourhoods; (ii) well-being, mental well-being, mental health, life satisfaction, happiness, quality of life, self-rated health, physical well-being, physical health, and healthy ageing; (iii) older people, older adults, older residents, and seniors. 

### 2.2. Study Inclusion

We identified the relevant publications according to the inclusion criteria. Eligible studies had to satisfy the following criteria: (i) Studies primarily investigated the associations between environmental components and outcomes of either physical or mental well-being of older adults; (ii) the measures of AFEs consisted of at least two environmental factors because this study aimed to estimate the combined effect of environments on physical and mental well-being; (iii) more than 50% of the participants were aged 60 years or older; conventionally, the WHO [1] and United Nations [34] commonly used the proportion of the individuals with chronological age of 60 or 65 years or over in the total population to reflect the ageing level of a society (iv) studies had to employ quantitative research methods; and (v) studies were written in English. 

### 2.3. Data Extraction and Quality Assessment

As shown in Table 1, the key information extracted from the selected studies included: (i) study information (author/(s), year of publication, period of study conducted, research site, ageing rate and urbanisation rate of local sites [35,36]); (ii) study characteristics (sample size, and age range of the participants); (iii) environmental components; (iv) outcomes of physical and mental well-being; and (v) a quality assessment of studies. 

A quality assessment of each study was conducted using an appraisal tool based on seven criteria [46,47], namely, (i) acceptable response rate of participants, (ii) representative sample size, (iii) valid and reliable measures of outcomes or key variables, (iv) adjustment for socio-demographic covariates, (v) appropriate analytical strategy, (vi) clear discussion in line with the results, and (vii) robustness and sensitivity tests. The assessment was independently conducted by the first and second author of this study. The two authors had a consistent rate of quality assessment of 86%, and discrepancies were further discussed and scrutinised to reach an agreement. The total score of each study was summed by the score of each criterion, and research quality was accordingly classified as high, moderate, or low. All the above-mentioned information was cross-checked to make sure of the data’s authenticity and reliability. 

### 2.4. Statistical Analysis

A random-effects model was used because the selected studies differed from each other in terms of research participants, sampling strategies, and research settings [48]. A random-effects model incorporated the variances into an overall estimated effect [49]. The raw data of the Pearson’s correlation *r* and the sample size of each study were used to estimate the pooled effect size of the association between combined environments and older adults’ physical and mental well-being. For studies where Pearson’s *r* and/or sample size were not reported in the published literature, we attempted to contact the corresponding author by email. The correlation was transformed through Fisher’s *z*, converting a standardised metric to be compared, with 95% confidence intervals (CIs) [48]. All the analyses were performed with Comprehensive Meta-Analysis 3.0 software (Biostat, Inc., Englewood, LA, USA) [50]. A positive correlation indicates that higher perceived scores of environments are correlated with better physical or mental well-being, and a negative correlation indicates the inverse. 

Because the effects are expected to be relatively consistent within a study, but substantially varied between studies, individual studies were used as the unit of analysis to estimate the between-study variation [48]. For studies involving more than one measure of the same outcome (e.g., measures of life satisfaction, meaning in life, and affection to represent mental well-being), the composite effect size for the correlation between these measures was computed. If an individual study examined more than one outcome variable (e.g., a measure for physical health and another measure for mental health), we used the number of outcomes to divide the sample size to reduce the impact on the overall effect size [51]. 

*Q* statistic was used to test heterogeneity and whether there was a true variance between groups of studies. In addition, we employed the *I*^2^ statistic to measure the proportion of the total variance of effect size attributable to between-studies variance [52]. There are some benchmarks for the value of *I*^2^, with 25%, 50%, and 75% presenting low, moderate, and high heterogeneity respectively [52]. Subsequently, a moderator analysis was conducted to examine whether the heterogeneity of the overall correlation could be moderated by the number of environmental components, sample size, ageing rate, and urbanisation rate of local research site. 

Lastly, we employed a funnel plot to visualise the presence of publication bias in the meta-analysis. As a supplement, Egger’s regression intercepts were used to provide more objective and precise information to identify publication bias [53]. 

## 3. Results

### 3.1. Characteristics of the Selected Studies

A total of 14 studies met the eligibility criteria and were included in the analysis. Among the selected studies, eight studies examined the relationship between AFEs and physical well-being, and eleven studies examined AFEs with mental well-being (five studies examined both aspects of well-being). Most studies (*n* = 10) were conducted during the period of 2015 to 2018. Correspondingly, all the studies were published from 2016 to 2021. This is because the empirical studies focusing on the relationship between AFEs and the well-being of older adults did not cluster until recent five years. Thirteen studies employed a cross-sectional research design, and one study used a longitudinal design [6]. For research sites, five studies were conducted in Hong Kong, two studies in New Zealand and the Netherlands, and the remaining studies were conducted in the United States, Spain, South Korea, Thailand, and mainland China. It is worth noting that most of the research settings were characterised by high ageing rate and urbanisation rate. For example, when the studies were conducted, 78% (*n* = 11) of research sites had a population ageing rate (share of people aged 65 and over) of 15% or higher, the universal criterion of identifying an “aged society” [34]. In addition, 64% (*n* = 9) of research sites had an urbanisation rate higher than 85% [35]. Participants aged 60 and over accounted for more than 50% of the participants in the selected studies, with the sample size ranging from 203 [37] to 4028 [5]. 

A variety of environmental components have been examined. The eight domains of the WHO’s age-friendly framework was most frequently measured among the selected studies [3,4,37,38,39,40,41]. The domains covered eight environmental factors, including outdoor spaces and buildings, housing, transportation, social participation, respect and social inclusion, civic participation and employment, communication and information, and community support and health services. In addition to the WHO’s framework, environmental components also encompassed access to community facilities, facility security, and activity centres for older adults, relations with residents, neighbourhood cohesion/trust, and crime-related neighbourhood safety. The number of environmental components involved in each study ranged from two [7] to eight [3,4,37,38,39,40,41]. Regarding the assessed outcomes of well-being, the studies showed that outcomes of physical well-being included self-rated health, physical comfort, and physical quality of life, while mental well-being consisted of outcomes such as life satisfaction, psychological or mental related quality of life, mental health, affection, active ageing and meaning in life. 

### 3.2. Associations between Combined Environments and Older Adults’ Physical and Mental Well-Being

**Figure 2:**
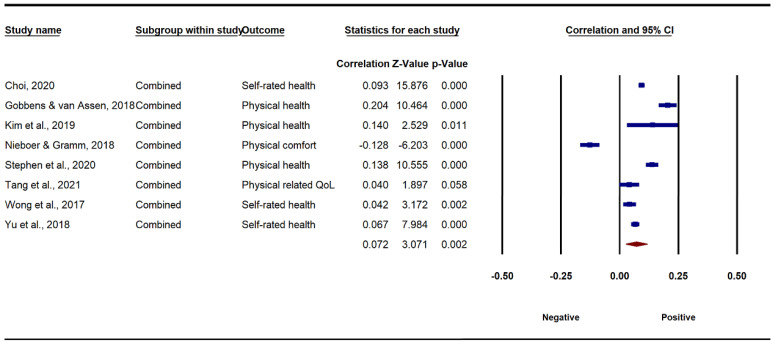
Forest plot of the association between AFEs and physical well-being.

**Figure 3:**
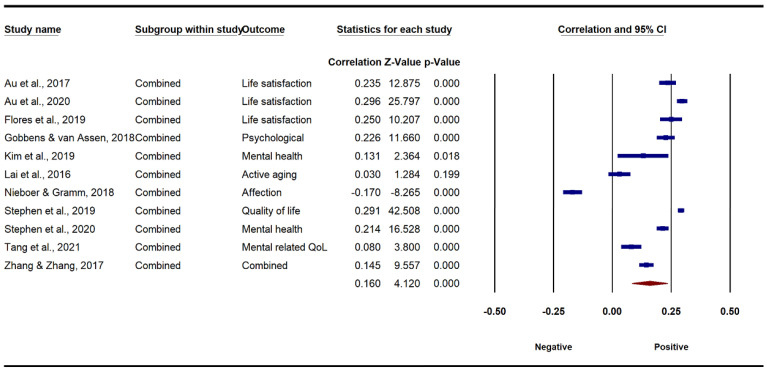
Forest plot of the association between AFEs and mental well-being.

Figure 2 and Figure 3 present the results of the associations between combined environmental components and older adults’ physical and mental well-being. Specifically, under the random-effects model, a small but significant association between combined environmental factors and older adults’ physical well-being was found (*r* = 0.072, 95% CI [0.026, 0.118], *p* < 0.01). A high degree of heterogeneity of the overall environment–physical well-being association among the studies was observed (*Q* = 182.76, *I*^2^ = 96.17, *p* < 0.001). The association between combined environmental components and older adults’ mental well-being was also significant, and relatively stronger (*r* = 0.160, 95% CI [0.084, 0.224], *p* < 0.001). Likewise, there was a considerable heterogeneity of the environment–mental well-being relationship (*Q* = 671.86, *I*^2^ = 98.51, *p* < 0.001). Generally, the results indicate that higher perceived ratings of environments were significantly and positively correlated with the physical and mental well-being of older adults. 

### 3.3. Moderator Analysis

Table 2 and Table 3 show the results of the moderator analyses for the outcomes of physical well-being and mental well-being. The analyses examined whether the number of environmental components, sample size, ageing rate, and urbanisation rate of research sites contributed to the heterogeneity of the correlation. As shown in Table 2, the association between environments and the physical well-being of older adults was significantly moderated by the number of environmental components (*Q_b_* = 5.665, *p* < 0.05). The person–environment relationship in studies that involved concentrated number of environmental components (*n* < 6) was significant (*r* = 0.130, 95% CI [0.065, 0.195], *p* < 0.05), while the association with broader environmental factors (*n* >= 6) was insignificant (*r* = 0.021, 95% CI [−0.040, 0.083], *p* > 0.05). It is also worth noting that the significance of associations in various sample sizes and ageing rates were also different, although the between-variance was not significant within the moderator group. 

In Table 3, the heterogeneity of the association between environments and mental well-being was not significantly attributed to the listed moderators. 

### 3.4. Publication Bias

**Figure 4:**
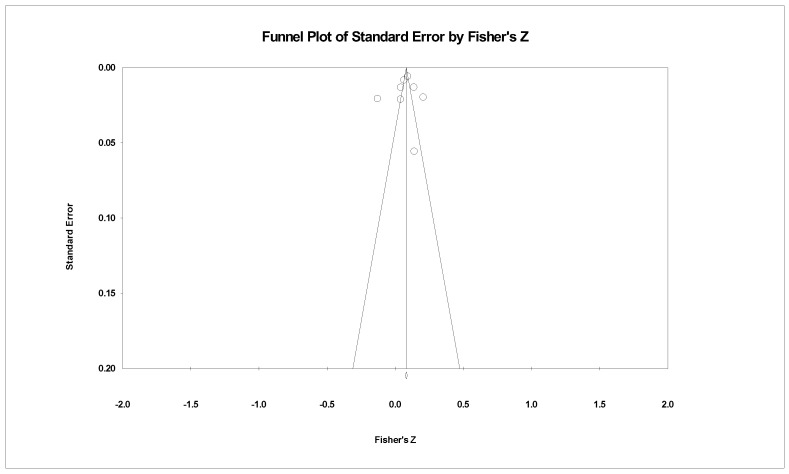
Funnel plot of the association between AFEs and physical well-being.

**Figure 5:**
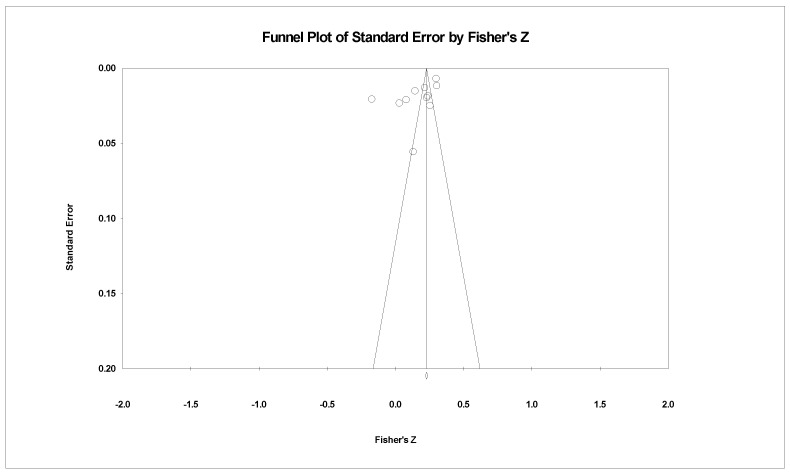
Funnel plot of the association between AFEs and mental well-being.

Two funnel plots were used to illustrate publication bias (Figure 4 and Figure 5), where the *x*-axis denotes Fisher’s *z* and the *y*-axis represents the standard error. In both funnel plots, the majority of the studies are distributed around the mean effect size at the top, although the symmetry is not visually apparent. Moreover, Egger’s test for the association between AFEs and the physical well-being of older adults (*b* = −1.420, 95% CI [−10.601, 7.760], *p* > 0.05) was not significant, indicating that publication bias was unlikely. However, Egger’s test indicated the likelihood of publication bias concerning the association between AFEs and mental well-being (*b* = −10.767, 95% CI [−20.941, −0.573], *p* < 0.05). This result implies that among the literature on the association of AFEs and mental well-being, studies that have discovered significant associations are more likely to be published [48]. 

## 4. Discussion

Based on the synthesised correlational results of the 14 quantitative studies, this meta-analysis provides more conclusive findings regarding the person–environment association. The analysis shows that combined environmental factors were significantly and positively associated with physical and mental well-being, although both associations were small. In addition, the considerable heterogeneity within the correlation between AFEs and physical well-being was significantly attributed to the number of environmental components. The findings of this meta-analysis also shed light on the implications for future empirical research in the field of environmental gerontology. The specific research findings and potential explanations are discussed below. 

First, this study summarised the characteristics of studies on the relationship between AFEs and older adults’ well-being. Among the selected studies, the majority employed a cross-sectional research design. This is understandable since the comprehensiveness of multiple environmental factors may become infeasible and cause cost-inefficiencies when using randomised experiments or longitudinal data. Moreover, driven by population ageing and urbanisation, the research sites of the selected studies were grouped in places with high population ageing rates and degrees of urbanisation. In terms of the instruments of AFEs, most studies adopted the widely accepted measures from the WHO’s framework of eight domains, while the remaining studies assessed alternative environmental factors from representative surveys. A wide range of physical and mental well-being outcomes was examined in the studies. Indicators of physical well-being included physical health, physical comfort, physical related quality of life, and self-rated health in selected studies. Measures of mental well-being encompassed life satisfaction, mental health, psychological and mental-related quality of life, meaning in life, and affection. 

Second, this meta-analysis discovered weak but significantly positive associations between AFEs and older adults’ physical well-being. The results of the present study not only confirmed the prominent association between older adults and combined environments but also quantified the magnitude of the association. The significant environmental influence on the physical well-being of older adults provided more robust evidence to challenge the inconsistent results among individual empirical studies. From the perspective of environmental gerontology, the fit or lack of fit between individual functional limitations in old age and external barriers was strongly linked to physical well-being, especially disability-related outcomes [27]. It is commonly believed that the perceptions of environments have a significant impact on the physical health of older adults by affecting the intensity of their participation in activities [54]. Admittedly, the strength of the association between AFEs and physical well-being was small. This might be explained by the observation that the environment may not be the most vital determinant of older adults’ physical well-being because genetic or biological determinants still play a substantial role in controlling and regulating individual physical levels [55,56].

The mean overall effect size of combined environments on older adults’ mental well-being was also significant. This finding resonated with the results of existing review studies where various environmental factors were salient predictors of mental health [32,33,57]. Researchers attempted to explain the significant link between environments and mental well-being from both direct and indirect pathways. In terms of the direct pathway, they illustrated that the perceptions and exposures of environmental characteristics could directly elevate positive emotions and affection, or ameliorate psychological stress [58]. Regarding indirect mechanisms, theoretical orientation and empirical evidence suggested that personal control, social support, and a sense of community may act as valid pathways linking environments and mental well-being [7,27,58]. 

Third, the current meta-analysis captured considerable heterogeneity in the associations and explored potential moderators for this heterogeneity. As indicated by previous studies [59,60], the large heterogeneity is not surprising because the selected studies varied widely in terms of research settings, sample size, and composition of environmental components. Previous review studies focusing on person–environment relationships also discovered large heterogeneity in selected empirical literature [29,61]. This research found that the number of environmental components significantly moderated the association between combined environments and the physical well-being of older adults. Particularly, the correlation between AFEs and physical well-being was only significant among studies with a concentrated number of environmental factors (*n* < 6). This finding reveals that compared with broader environmental compositions, specific vital environmental factors may generate a prominent influence on older adults’ physical well-being. It suggests the importance of identifying core environmental components in creating AFEs by incorporating with local contexts and the needs of older adults. 

## 5. Limitations 

Several limitations of this study need to be noted. First, this study only examined the effect size of combined environments on promoting positive well-being outcomes in older adults. The extent to which environments may prevent negative well-being outcomes has not been investigated due to the limited number of studies focusing on unfavourable outcomes. Second, AFEs measures may not be absolutely objective since all the selected studies assessed environmental components by using subjective self-perceptions, which may influence the precision of measurements. Third, the results in this meta-analysis could not conclude causal inferences of the association between environments and well-being because all the selected studies (except Stephens et al., 2020) employed a cross-sectional research design. 

## 6. Implications

Given the above-mentioned limitations of extant literature, this study proposed the following suggestions for future research and practice. First, in addition to the positive outcomes, studies could explore the influence of AFEs on adverse outcomes of physical well-being (e.g., functional decline, frailty, and falls) and mental well-being (e.g., cognitive impairment, anxiety, or depression). Second, more objective measures by adopting technics (e.g., geographic information system, google mapping) should be applied in future studies to provide a more accurate assessment of environmental factors and specially built environments. Third, more rigorous study designs (e.g., quasi-experimental design) are suggested to further determine whether AFEs and older adults’ well-being are causally related. Fourth, as a result of the significant moderator of the number of environmental components, specific environmental factors or types should be identified to capture a more robust estimation of the outcome of physical well-being. Fifth, a perspective of socioeconomic stratification should be applied to future studies to examine the influence of environments on older adults. It is likely that exposure to different environmental conditions is not randomly distributed, but clustered in alignment with corresponding socio-economic levels. Thus, older residents’ socio-economic background or residence affordability should be taken into consideration in future studies. 

In line with the Decade of Healthy Ageing: 2020–2030 [2], researchers and practitioners have started to launch broader solutions to promote healthy ageing and equality through neighbourhood projects. This meta-analytical study can make a few suggestions for policy and program planning. First, environmental interventions should be regarded as an effective means to promote well-being, especially mental aspects, among community-dwelling older adults. Thus, a combination of age-friendly environmental components should be developed for older adults, particularly for those with a higher risk of experiencing mental disorders. Second, to identify the salient components and reduce the heterogeneity of effect size, policymakers should conduct a preliminary investigation to examine the key demand for environmental factors among older residents. Moreover, as suggested by the pathways linking individuals and their environment, community activities or programs should be designed that is tailored to the needs or characteristics of older residents living in diverse communities. 

## 7. Conclusions

Consistent with the research objectives, this study contributed to knowledge building in the field of ageing and environment in the following three ways. First, it summarised characteristics regarding research sites, research design, involved environmental components, and well-being outcomes of the studies focusing on person–environment relationships. Second, this meta-analysis synthesised a small but significant correlation between AFEs and older adults’ mental and physical well-being. This result supported the significant person–environment relationship as suggested by the theory of environmental gerontology. Furthermore, by providing the precise magnitude of the association of environment–physical well-being and environment–mental well-being, this study addressed the inconclusive findings in individual empirical studies and general review studies. Particularly, the effect size of AFEs was slightly stronger on mental well-being, than on physical well-being. This indicates that AFEs may be more effective in promoting positive emotions or feelings in older adults, rather than individual functional ability. Third, considerable heterogeneity existed in both correlations of environment–physical well-being and environment–mental well-being. The association between AFEs and physical well-being was significantly moderated by the number of environmental factors, implying the priority to identify the specific environmental components that are vital for older adults in future research and practice.

## Figures and Tables

**Table 1:** Summary of information of selected studies.

Author (Year)	Period of Study Conducted	Research Site	Ageing Rate	Urbanisation Rate	Sample Size	Age Range	Environmental Components	Outcomes of Well-Being		Quality Assessment
								Physical Well-Being	Mental Well-Being	
Au et al., 2020 [3]	2016	Hong Kong	16%	100%	898	55–97	Outdoor spaces and buildings; Housing; Transportation; Community services *; Social participation *; Communication and information; Respect and social inclusion; Civic participation and employment		Life satisfaction	Moderate
Au et al., 2017 [4]	ns	Hong Kong	15%	100%	682	65–97	Outdoor spaces and buildings; Housing; Transportation *; Community services; Social participation *; Communication and information; Respect and social inclusion; Civic participation and employment		Life satisfaction	Moderate
Stephens et al., 2019 [5]	2016	New Zealand	15%	86%	4028	50–89	Housing *; Neighbourhood accessibility *; Distance to health services; Neighbourhood cohesion *; Neighbourhood safety/security		Quality of life	Moderate
Stephens et al., 2020 [6]	2016, 2018	New Zealand	16%	87%	2910	60–70+	Housing; Access to facilities *; Neighbourhood trust *; Neighbourhood safety/security	Physical health	Mental health	Low
Tang et al., 2021 [7]	2015, 2016, 2017	Hong Kong	16%	100%	2247	65–79	Outdoor spaces *; Outdoor buildings *	Physical related QoL	Mental related QoL	High
Flores et al., 2019 [37]	ns	Spain	19%	80%	203	60–75	Outdoor spaces and buildings *; Housing; Transportation; Social participation; Communication and information; Respect and social inclusion; Civic participation and employment; Community services *		Life satisfaction	Low
Yu et al., 2018 [38]	2015–2017	Hong Kong	16%	100%	1798	60–80+	Outdoor spaces and buildings *; Housing *; Transportation *; Community services; * Social participation; Communication and information; Respect and social inclusion *; Civic participation and employment	Self-rated health		Moderate
Nieboer & Gramm, 2018 [39]	2013	Netherlands	17%	89%	588	70–93	Outdoor spaces and buildings *; Housing *; Transportation *; Social participation *; Communication and information *; Respect and social inclusion *; Civic participation; Community services *	Physical comfort	Affection	High
Wong et al., 2017 [40]	2015	Hong Kong	15%	100%	719	60–80+	Outdoor spaces and buildings *; Housing *; Transportation *; Community services; Social participation *; Communication and information; Respect and social inclusion *; Civic participation and employment	Self-rated health		Low
Choi, 2020 [41]	2015	United States	15%	82%	3650	65–101	Outdoor spaces and buildings *; Housing; Transportation *; Social participation; Communication and information; Community services; Volunteer and civic engagement; Job opportunities	Self-rated health		High
Gobbens & van Assen, 2018 [42]	2009–2010	Netherlands	15%	87%	1031	65–95	Outdoor spaces *; Housing *; Transportation; Community facilities *; Relationships with residents *	Physical related QoL	Psychological related QoL	Moderate
Kim et al., 2019 [43]	2017	South Korea	14%	82%	651	50–70+	Safe roads; Transportation; Community services; Social participation; Respect and social inclusion	Physical health	Mental health	Moderate
Lai et al., 2016 [44]	ns	Thailand	10%	46%	613	45–65+	Outdoor spaces and buildings; Transportation and housing; Community services		Active aging	Moderate
Zhang & Zhang, 2017 [45]	2015	China	9%	56%	720	50+	Outdoor spaces and buildings *; Transportation *; Access to facilities *; Facility security *; Activity centers *; Community services *		Life satisfaction; Meaning in life; Affection	High

Note. Ageing rate was measured by the proportion of population aged 65 and over accounting for the total population of each research site when the study was conducted, and data came from The World Bank https://data.worldbank.org/indicator/SP.POP.65UP.TO.ZS (accessed on 6 August 2022); Urbanisation rate was measured by the percent of urban population of each research site when the study was conducted, and the data were drawn from The World Bank https://data.worldbank.org/indicator/SP.URB.TOTL.IN.ZS (accessed on 6 August 2022); For those studies that did not state when the research was conducted, the publication year minus three years was used as the year to extract local ageing rate and urbanisation rate for; In the study of Lai et al. (2016), the outcome of active ageing was a composite variable measured by the sum of the quality of life, well-being, and life are good; ns = not stated; QoL = quality of life; * denotes the result showed that the influence of environmental component is significant on well-being outcomes in each individual study.

**Table 2:** Moderator analysis of the association between AFEs and physical well-being.

Moderator Group	Subgroup	*k*	*r*	95% CI	*Q_b_*
Number of environmental components	<6	4	0.130	[0.065, 0.195]	5.665 *
	> = 6	4	0.021	[−0.040, 0.083]	
Sample size	<1000	4	0.058	[−0.012, 0.128]	0.307
	>1000	4	0.085	[0.019, 0.150]	
Ageing rate of research site	< = 15%	4	0.117	[0.040, 0.194]	2.455
	>15%	4	0.031	[−0.044, 0.106]	
Urbanisation rate of research site	< = 85%	2	0.112	[−0.012, 0.233]	0.502
	>85%	6	0.061	[−0.006, 0.128]	

Note. * *p* < 0.05.

**Table 3:** Moderator analysis of the association between AFEs and mental well-being.

Moderator Group	Subgroup	*k*	*r*	95% CI	*Q_b_*
Number of environmental components	<6	6	0.165	[0.053, 0.272]	0.017
	> = 6	5	0.154	[0.033, 0.271]	
Sample size	<1000	8	0.145	[0.050, 0.238]	0.331
	>1000	3	0.197	[0.045, 0.340]	
Ageing rate of research site	< = 15%	6	0.179	[0.068, 0.286]	0.260
	>15%	5	0.137	[0.015, 0.254]	
Urbanisation rate of research site	< = 85%	4	0.140	[0.012, 0.264]	0.148
	>85%	7	0.171	[0.077, 0.262]	

## Data Availability

The data presented in this study are available on request from the first author.
